# Optogenetic engineering of synthetic and natural receptors: design principles, functional mechanisms and biomedical applications

**DOI:** 10.1093/rb/rbaf126

**Published:** 2025-12-17

**Authors:** Jiaying Zhao, Yani Chen, BiCong Gao, Lujiao Zhang, Ning Gao, Sijia Hao, Zili Gao, Wenjin Cai, Jian Yang, Guoli Yang

**Affiliations:** Stomatology Hospital, School of Stomatology, Zhejiang University School of Medicine, Zhejiang Provincial Clinical Research Center for Oral Diseases, Zhejiang Key Laboratory of Oral Biomedical, Hangzhou 310000, PR China; Stomatology Hospital, School of Stomatology, Zhejiang University School of Medicine, Zhejiang Provincial Clinical Research Center for Oral Diseases, Zhejiang Key Laboratory of Oral Biomedical, Hangzhou 310000, PR China; Stomatology Hospital, School of Stomatology, Zhejiang University School of Medicine, Zhejiang Provincial Clinical Research Center for Oral Diseases, Zhejiang Key Laboratory of Oral Biomedical, Hangzhou 310000, PR China; Department of Materials Science and Engineering, Westlake University, Hangzhou, Zhejiang 310030, PR China; Research Center for Industries of the Future, Westlake University, Hangzhou, Zhejiang 310030, PR China; Stomatology Hospital, School of Stomatology, Zhejiang University School of Medicine, Zhejiang Provincial Clinical Research Center for Oral Diseases, Zhejiang Key Laboratory of Oral Biomedical, Hangzhou 310000, PR China; Stomatology Hospital, School of Stomatology, Zhejiang University School of Medicine, Zhejiang Provincial Clinical Research Center for Oral Diseases, Zhejiang Key Laboratory of Oral Biomedical, Hangzhou 310000, PR China; Department of Materials Science and Engineering, Westlake University, Hangzhou, Zhejiang 310030, PR China; Research Center for Industries of the Future, Westlake University, Hangzhou, Zhejiang 310030, PR China; Stomatology Hospital, School of Stomatology, Zhejiang University School of Medicine, Zhejiang Provincial Clinical Research Center for Oral Diseases, Zhejiang Key Laboratory of Oral Biomedical, Hangzhou 310000, PR China; Department of Materials Science and Engineering, Westlake University, Hangzhou, Zhejiang 310030, PR China; Research Center for Industries of the Future, Westlake University, Hangzhou, Zhejiang 310030, PR China; Center for Biobased Materials, Muyuan Laboratory, Zhengzhou, Henan Province 450016, PR China; Stomatology Hospital, School of Stomatology, Zhejiang University School of Medicine, Zhejiang Provincial Clinical Research Center for Oral Diseases, Zhejiang Key Laboratory of Oral Biomedical, Hangzhou 310000, PR China

**Keywords:** optogenetic receptor engineering, biomedical applications, synthetic receptors, intracellular signaling, reversible control

## Abstract

Cellular receptors serve as central hubs that translate external signals into intracellular programs governing cell fate, function and behavior. Achieving precise and reversible control over receptor activity has long been a major challenge in both fundamental biology and translational medicine. Optogenetic receptor engineering provides a transformative solution by integrating photosensitive domains into natural receptor frameworks. This strategy enables light-dependent modulation of signaling with high spatial and temporal precision while maintaining minimal disturbance to endogenous pathways. Unlike chemogenetic systems or classical photoreceptive ion channels, this approach preserves endogenous ligand specificity and avoids slow ligand diffusion/clearance-associated artifacts. Through such systems, researchers can dissect causal relationships in dynamic signaling events, finely manipulate neuromodulatory and immune circuits and program cellular activities involved in development and tissue regeneration. The approach also allows quantitative control of signaling intensity and duration, offering new opportunities for linking molecular design to physiological outcomes. By combining optogenetic principles with advances in materials science and bioelectronics, future designs may achieve improved optical fidelity, enhanced light penetration and better signal amplification within complex biological environments. Integration with AI-guided protein engineering may also accelerate the discovery of optimized photosensory–receptor pairings. Together, these developments point to an emerging field where light-responsive receptors function as programmable interfaces between photonic control and cellular computation. In summary, the engineering of optogenetic receptors establishes a conceptual and technological framework for reversible, accurate and tunable regulation of cellular communication. This review summarizes current progress, outlines key design principles and provides conceptual guidelines for advancing next-generation light-responsive receptors and their biomedical applications. However, key translational challenges—including immunogenicity of non-human photoreceptors, limited gene-delivery efficiency and long-term biosafety—remain to be addressed through nonviral delivery strategies, autologous cell engineering and de-immunized or humanized photoreceptor design.

## Graphical abstract

**Figure:**
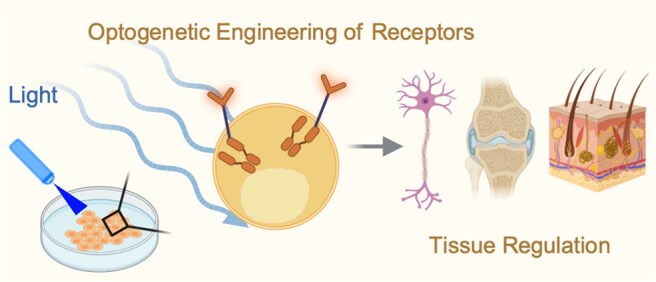

## Introduction

### Central role of natural receptors and challenge of precision perturbation

Cell surface and intracellular receptors, such as G protein-coupled receptors (GPCRs), receptor tyrosine kinases (RTKs) and cytokine receptors, represent the major class of regulators of cellular responses. They transduce the extracellular signal into distinct intracellular pathways that control cell fate, function and behavior [1, 2]. However, traditional tools, including pharmacology and genetic knockout, could not capture fast, localized and dynamic signals, which for a long time has remained a challenge to precisely dissect these signaling networks [3]. This is because most of the currently available pharmacological agents generally act slowly and often promiscuously, and genetic perturbations are irreversible, which generally leads to compensatory adaptations in cells that obscure direct causality [4].

### The evolution of control: from chemogenetics to optogenetics

Chemogenetic tools, such as Designer receptors exclusively activated by designer drugs (DREADDs), have improved cellular specificity but remain constrained by ligand pharmacokinetics, providing limited temporal resolution and minimal spatial control [5–7]. In contrast, classical optogenetic tools (e.g. channelrhodopsins) revolutionized neuroscience by enabling millisecond-precision control of neuronal firing [8]. However, these opsins primarily regulate electrical activity and are not naturally suited for controlling complex biochemical signaling cascades downstream of most endogenous receptors [9, 10]. This gap has been progressively filled by the discovery and engineering of diverse photoreceptor modules (e.g. Light-Oxygen-Voltage [LOV], Cryptochrome 2/Cryptochrome-Interacting Basic-helix-loop-helix [CRY2/CIB], Phytochrome B/phytochrome interacting factor [PhyB/PIF]), which allow optical control of protein–protein interactions, conformation and localization with high spatiotemporal fidelity [7, 11, 12].

### Optogenetic engineering: grafting a light-switch onto native receptors

This review focuses on a strategy for the optogenetic engineering of natural receptors. This approach retains the native architecture and signaling logic of receptors while equipping them with a photonic control interface [13]. By embedding light-sensitive domains into natural receptor frameworks, researchers can now use light to dictate when, where and to what extent a signaling pathway is activated, with minimal interference to its inherent biological fidelity [8, 14]. Key engineering strategies have mainly fallen into the following categories:

1. Light-controlled allosteric regulation: light-modulating LOV domain by some effects leads to masking or releasing the active site of the protein itself [15–18];
2. Optical reconstruction: Using photo-induced dimers—such as CRY2/CIBN, iLID/SspB—to reconstitute split receptor domains for downstream signaling [19–21];
3. Phototranscriptional control: Application of receptor function to gene expression by red light system (like PhyB/PIF) improves the tissue penetration ability [22];
4. Native optoGPCRs: naturally photosensitive GPCRs, such as PdCO, directly and efficiently couple to the endogenous Gi/o pathways [23, 24].

Most traditional synthetic receptors are based on irreversible proteolysis or fixed transcriptional output [8, 25, 26]. By contrast, receptors modified by optogenetic engineering can precisely control endogenous signal dynamics in a reversible, quantitative and region-specific manner. This might be contributing to the reduction of off-target effects and be favorable for studying the causality between physiology and disease mechanisms [7, 27, 28]. Compared with traditional chemogenetic systems that require synthetic ligands with slow diffusion and clearance kinetics, optogenetic engineering preserves endogenous ligand–receptor architecture and avoids pharmacokinetic artifacts [29, 30]. Unlike classical photoreceptive ion channels (e.g. channelrhodopsins), which mainly alter membrane voltage rather than native signaling cascades, optogenetic receptors modulate the intrinsic biochemical pathways of GPCRs, RTKs and cytokine receptors with high precision [31, 32].

**Figure 1:**
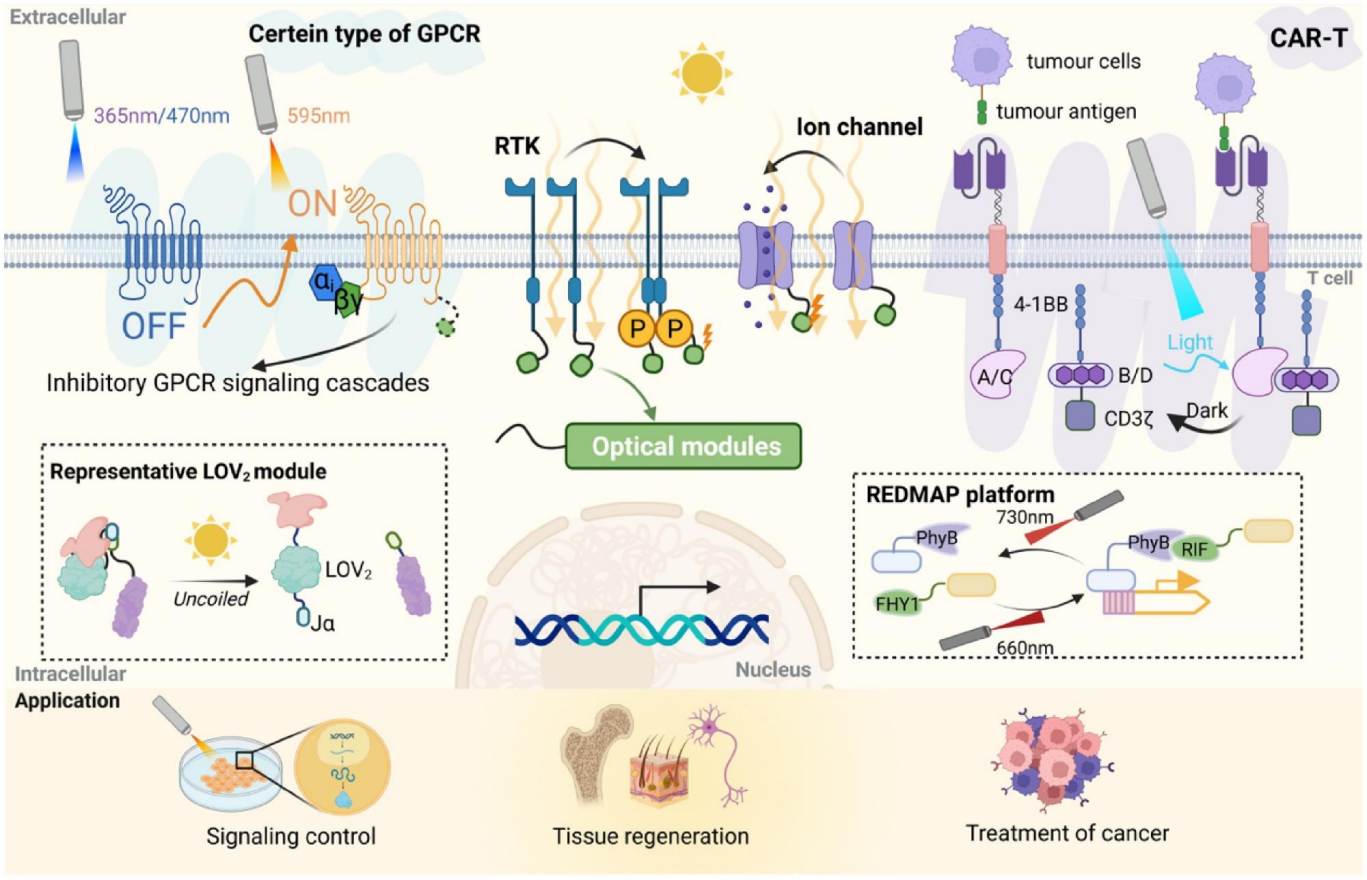
Schematic overview of optogenetic receptor engineering. Natural receptor systems (GPCRs, RTKs and ion channels) can be reprogrammed with light-responsive modules (LOV modules, REDMAP platform, etc.) to achieve spatiotemporally precise signaling control across cellular, tissue and therapeutic contexts.

First, we systematically classified these engineering strategies (Design Strategies for the Optogenetic Engineering of Receptors Section), and further elaborated on their implementation methods in key receptor families and their applications in fields such as immunology and neuroscience (Major Types and Representative Systems of Optogenetically Engineered Receptors Section) [33, 34]. Finally, the current persistent challenges are discussed, such as dark state leakage, spectral orthogonality and *in vivo* light delivery issues (Applications of Optogenetically Engineered Receptors Section) [22, 35–37]. This review aims to provide researchers with tools for precisely controlling cell signals using light, and to open up new frontiers in basic research and therapeutic intervention (Figure 1).

## Design strategies for the optogenetic engineering of receptors

Optogenetic engineering is capable of regulating receptor-mediated signal transduction, and the precision of this regulation in terms of time and space was previously unachievable. By embedding the photosensitive domain into specific receptor elements, researchers were able to introduce reversible and quantitative external control while retaining the endogenous signal transduction structure [8, 14]. In actual design, the design content of optogenetic receptors is usually divided into the following four steps: optical sensing module, receptor scaffold, coupling interface and verification strategy [38, 39].

### Design elements: photosensory modules, receptor targets, coupling interfaces and validation

Selection and configuration of the photosensory module form the very backbone of every optogenetically engineered receptor. Various commonly used domains include the LOV domain from Avena sativa phototropin, the CRY2/CIBN pair from Arabidopsis thaliana and the PhyB/PIF6 system [12, 20, 40, 41]. Emerging modules, such as Cyanobacteriochromes (CBCRs), expand the accessible spectral range into the red and near-infrared regions [42]. Recent advances have introduced additional deep-tissue-compatible modules, such as engineered BphP1–QPAS1 systems that respond to near-infrared light, upconversion nanoparticle–assisted activation and bioluminescent optogenetic systems (e.g. NanoLuc–LOV hybrids), which enable receptor activation without external illumination and broaden the translational feasibility of optogenetic receptors [32, 43].

**Figure 2:**
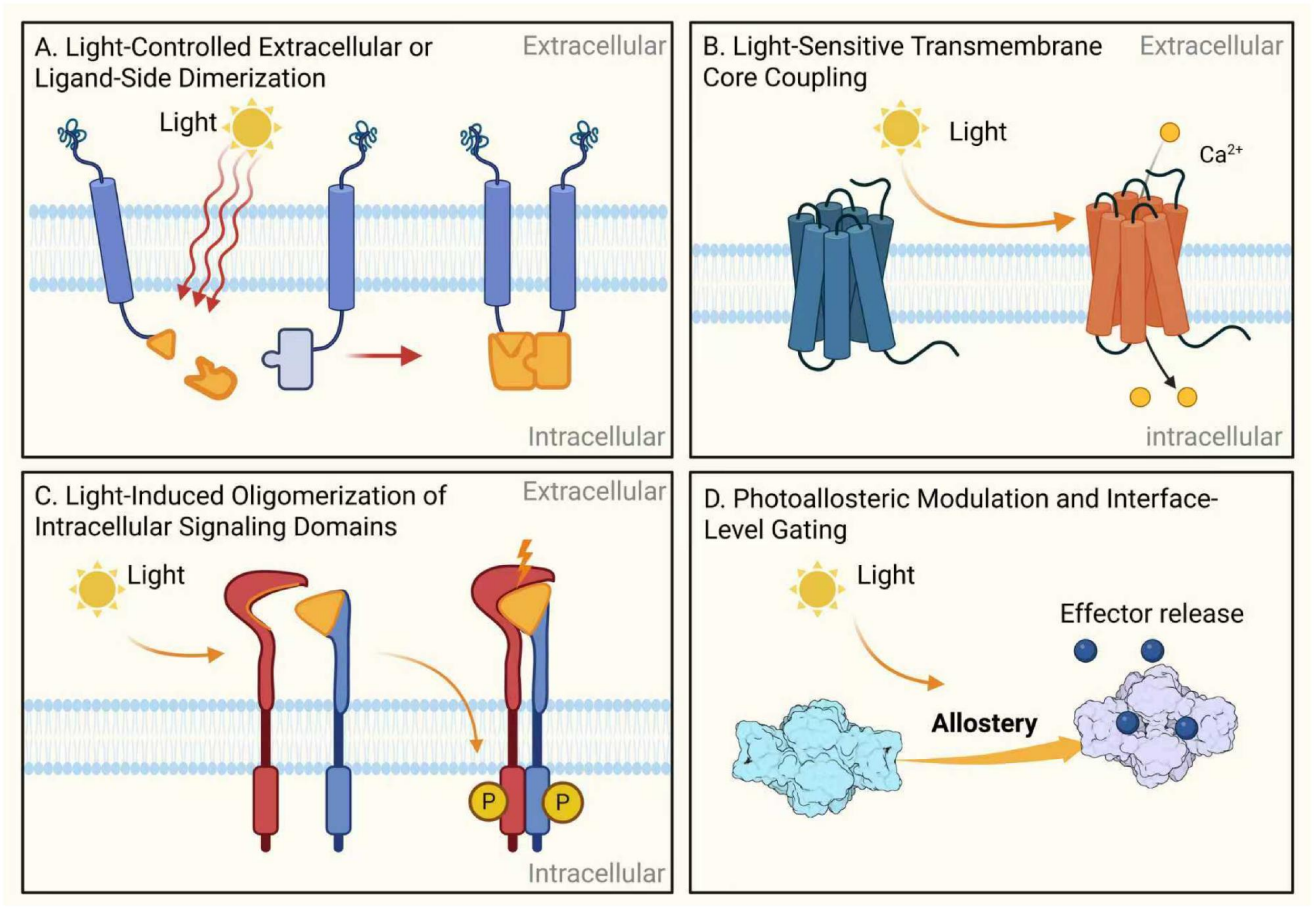
Representative strategies for optogenetic receptor engineering. Modular design approaches include (**A**) extracellular or ligand-side photodimerization, (**B**) light-sensitive transmembrane coupling, (**C**) intracellular light-induced oligomerization and (**D**) allosteric and transcriptional interface gating.

In addition to photosensory domain selection, the rational engineering of receptors involves four greater, interrelated aspects that collectively predestine performance and translational robustness. The design/strategy, therefore, includes: (i) light sensors and cofactors, (ii) receptor scaffold selection, (iii) coupling-interface design and (iv) functional validation (Figure 2).

- Light Sensors and Cofactors: Different photosystems have different activation spectra, kinetics and cofactor dependencies [44]. For example, PhyB- and CBCR-based systems utilize the chromophore phycocyanobilin (PCB), which likely has to be supplied exogenously or biosynthesized *in vivo* [45, 46]. LOV and CRY2, on the other hand, are flavin-based and largely self-sufficient in most mammalian cells [47].
- Receptor Scaffold Selection: GPCRs, RTKs, ligand-gated ion channels and cytokine receptors serve as natural templates [1, 48, 49]. The goal is to maintain the structural integrity and signaling specificity of the native receptor while introducing a photoresponsive gate.
- Coupling Interface Design: Photomodules can be coupled to extracellular, transmembrane or intracellular receptor regions. The interconnecting linker sequence is crucial, usually short, 5–20 amino acids [50] and needs to be optimized for the trade-off between conformational freedom and efficient allosteric transmission. Membrane topology and protein trafficking concerns have to be cautiously taken into consideration [51].
- Functional Validation: Extensive functional characterization needs to confirm the following: (1) least/no activity in the dark state (low leak) [51], (2) strong and reversible light-dependent activation, (3) signaling fidelity—meaning maintenance of ligand bias or kinetics of the action compared to the natural pathway, and finally, (4) proper subcellular localization [21, 24, 52]. Some of the conventional approaches include FRET-based biosensors, phospho-specific protein assays and live-cell imaging [8, 29].

While all four engineering strategies successfully enable optical modulation of receptors, they exhibit distinct characteristics and trade-offs:

- **Extracellular dimerization (Strategy I)**: Physiologically intuitive and mimics ligand-induced clustering, but photodimerizers are large, and steric interference with native ligand binding may occur [32].
- **Transmembrane or rhodopsin-based systems (Strategy II)**: Provide fast GPCR-like response kinetics, but rely on retinal availability and may impose topological constraints on receptor domains [53].
- **Light-induced oligomerization (Strategy III)**: Highly modular and excellent for clustering-dependent receptors such as RTKs and chimeric antigen receptor (CARs); however, the oligomerization stoichiometry can vary, affecting signaling uniformity [32, 53].
- **Photoallosteric regulation (Strategy IV)**: Enables fine-grained conformational control without major structural remodeling, yet requires empirical optimization of insertion sites to avoid misfolding or loss of function [54, 55].

These distinctions highlight the need to align strategy selection with receptor topology, intended signaling logic and target biological applications.

### Strategy I: light-controlled extracellular or ligand-side dimerization

Optical control can be exerted at the extracellular interface to substitute for ligand-induced dimerization [14, 56]. These optical dimerizers have been used as fusion modules with target proteins to drive light-induced interactions. For example, PhyB/PIF and LOV domains have been widely used to drive the membrane recruitment of proteins [57, 58]. In principle, such modules can be grafted onto receptor ectodomains to drive light-triggered pairing at the cell surface. Alternatively, photolabile or photoisomerizable groups can be attached to native agonists to design a photoactivatable ligand, thus, creating a “caged-ligand” system [35]. Such strategies are particularly relevant to receptor systems, whose activation relies on precise dimer geometry, such as members of cytokine and death receptor families, where ligand-induced clustering determines downstream signaling fidelity [59–62]. Irrespective of the strategies reviewed above, most optogenetic clustering approaches have remained proof-of-concept. Yet they illustrate that extracellular light can emulate critical aspects of ligand-driven activation with high temporal precision [63]. However, such extracellular strategies face specific constraints:

- dependence on exogenous cofactors—e.g. PCB for PhyB systems [64],
- potential interference with endogenous ligand binding and receptor trafficking [65],
- poor tissue penetration of short-wavelength actuators [12, 66].

Despite these challenges, light tunable ligands and ectodomain fusions offer a complementary route for optical control when preserving the native intracellular structure and the signaling complex is paramount [20].

### Strategy II: light-sensitive transmembrane core coupling (the Opto-XR paradigm)

The Opto-XR design pioneered a widely used route to light-controlled GPCRs by replacing the native transmembrane core of a target receptor with that of rhodopsin, a naturally light-activated GPCR [29, 53, 67]. This chimeric approach preserves the intracellular signaling loops of the original receptor while conferring light sensitivity via retinal-driven activation of rhodopsin [29, 68]. Upon light, the bound retinal undergoes 11-cis to all-trans isomerization, driving conformational changes that allow the retained cytoplasmic loops to interact selectively with specific G proteins [69–71]. Hence, by choosing appropriate intracellular domains, the engineered receptor subtype can achieve selective recruitment of Gs, Gi or Gq, which represent the stimulatory, inhibitory and phospholipase-C-beta (PLCβ)-coupled branches of heterotrimeric G-protein signaling, thereby enabling tailored downstream pathway activation [29, 53, 72]. Examples include the chimeric rhodopsin/β2 adrenergic receptor (Opto-β_2_AR) and the optogenetically engineered α_1_-adrenergic receptor (Opto-α_1_AR) [68, 73–75], which in a light-dependent manner robustly activates cAMP or PLC signaling, respectively, with their specificity dictated by their engineered intracellular regions.

This paradigm enables rapid and temporally precise receptor photoactivation [68, 76]. Measurable second-messenger outputs typically rise on sub-second to second timescales [68, 77]. This approach has been widely used to dissect pathway kinetics, desensitization and aspects of signaling bias [78, 79]. However, outstanding challenges include reliance on retinal as a cofactor and the need for correct topological alignment between transmembrane domains [77, 80]. Accordingly, engineering has optimized linker geometry and applied mutational tuning to enhance signal to noise, reduce dark activity and maintain correct expression and localization [53].

### Strategy III: light-induced oligomerization of intracellular signaling domains

This class of designs utilizes light-induced dimerization or clustering of receptor intracellular domains to recapitulate activation normally triggered by ligand binding. In such systems, light-responsive pairs such as CRY2/CIBN or improved Light-Inducible Dimer/Stringent starvation protein B (iLID/SspB) are fused to the cytoplasmic kinase or adaptor modules of RTKs, enabling optical control over receptor oligomerization and subsequent autophosphorylation [18].

In early proof-of-concept studies, researchers fused photosensitive modules (e.g. CRY2) to the intracellular domain of Tropomyosin receptor kinase A (TrkA). This strategy enables light-induced receptor clustering, thus, driving downstream signaling. The activated pathways included mitogen-activated protein kinases (MAPK) and phosphatidylinositol 3-kinase/protein kinase B (PI3K/Akt). Importantly, this downstream activation was rapid, occurring in seconds to minutes [81]. Similar strategies have been extended to other RTKs; near-infrared-responsive constructs, including Danio rerio Tropomyosin receptor kinase A (Dr-TrkA) and Danio rerio Tropomyosin receptor kinase B (Dr-TrkB), allow for reversible and deep-tissue control of neurotrophin receptor signaling [82, 83]. More recently, an optogenetic probe, termed OptoT1s, was engineered to modulate TrkB.T1, the truncated dominant-negative isoform of TrkB receptor, via light-induced clustering, further illustrating intracellular oligomerization as a versatile mode of activation [84]. Correct membrane localization is critical and is often achieved by tethering of the cytosolic modules with minimal transmembrane anchors or lipidation motifs to mimic native receptor geometry [85–88].

The Opto-XR strategy redesigns the transmembrane core of GPCRs. In contrast, the light-induced oligomerization approaches only have interference with the intracellular interface. This alternative design preserves the native extracellular and transmembrane regions of the receptor [32, 76, 88]. Such a strategy makes them broadly applicable to receptor classes whose activation has strong dependence on the clustering of the cytosolic domain rather than on transmembrane conformational transmission [88]. It has also been effectively translated into synthetic receptor frameworks, most notably in optogenetically CAR-T-cell systems [18, 89]. For instance, the LiCAR-T platform splits a conventional CAR into two modules, with one containing the antigen-binding domain and the other containing intracellular signaling motifs such as CD3ζ, each fused to complementary photodimerizing pairs (e.g. CRY2/CIBN or LOV_2_-based iLID/SspB). Then, blue-light illumination drives the heterodimerization of these two modules at the plasma membrane, to reconstitute a functional signaling complex enabling precise, reversible control of T-cell activation and cytotoxicity [34]. By this modular paradigm, optogenetic receptor engineering principles can be applied to create programmable and tunable cellular immunotherapies.

### Strategy IV: photoallosteric modulation and interface-level gating

In this category, light regulates receptor function through allosteric coupling or exposure of cryptic signaling motifs, rather than direct physical dimerization [90, 91]. By inserting LOV2 or similar modules into flexible loops of receptor intracellular or linker regions, light-induced conformational rearrangement toggles the accessibility of active sites or protease cleavage sequences [33, 92, 93].

Representative systems, including iTango, Cal-Light and FLiCRE, integrate this concept with intracellular signaling intermediates such as β-arrestin or Ca^2+^-dependent enzymes. This integration creates a logical “AND” gate for transcriptional responses. Gene expression is initiated only when both receptor activation and illumination coincide [33, 93].

The REDMAP platform extends these principles into the red-light regime by employing PhyB-PIF6-mediated reversible dimerization to control transcription factor nuclear entry and promoter activation [22]. Collectively, these interface-level gates effectively bridge transient receptor activity to programmable genomic outputs with optical precision.

Several innovative and powerful tools in optogenetics, such as PhoCl, AzoF and OptoNotch, operate outside the canonical receptor framework. Their functions are categorized as either nonreceptor-level release systems or *de novo* synthetic constructs. Therefore, they are discussed separately as conceptual inspirations for future design rather than as existing approaches of native receptor engineering [94–96].

A summary of the optical and biophysical characteristics of commonly used photosensory modules relevant to these strategies is provided in Table 1.

**Table 1:** Optical and biophysical parameters of representative photosensory modules.

Photosensory module	Activation wavelength	Dark recovery half-time	Mechanism	Cofactor requirement	Representative engineered systems
LOV2	450–470 nm	5–20 s	Jα-helix unfolding/allosteric gating	FMN (endogenous)	LOV2-inserted GPCRs, Cal-Light
CRY2/CRY2-CIB	450–488 nm	1–6 min	Oligomerization/CRY2-CIB docking	FAD (endogenous)	Opto-FGFR, Opto-iTrkB, OptoCAR
PhyB-PIF	630 nm (On), 740 nm (Off)	Seconds–minutes	Reversible photodimerization	PCB (exogenous or biosynthesized)	Red-light RTKs, Cytokine receptor mimetics
BphP1-QPAS1	740–780 nm	1–5 s	NIR-regulated docking	Biliverdin (endogenous in mammals)	Deep-tissue RTKs, NIR optogenetics
UCNP-assisted systems	808/980 nm → blue-light emission (upconversion)	N/A	Indirect nanoparticle-mediated activation	None	Wireless in vivo activation (e.g. OptoCAR-T)

## Major types and representative systems of optogenetically engineered receptors

Optogenetic receptor engineering has been successfully applied to diverse receptor families, including GPCRs, RTKs, ligand- and cytokine-gated receptors and ion channels [60, 97–99]. Each receptor type employs one or more of the design strategies outlined in Design Strategies for the Optogenetic Engineering of Receptors Section, while preserving the intrinsic signaling logic of its native counterpart. The following subsections summarize representative systems, their molecular mechanisms, and the principles used to ensure accurate optical control and physiological relevance.

### Light-activated GPCRs (primarily strategy I: transmembrane core coupling)

Light-controlled GPCRs, known as Opto-XRs, represent one of the earliest and most established paradigms in optogenetic receptor engineering [100]. These chimeric receptors replace the native seven-transmembrane (7TM) domain of a target GPCR with that of bovine rhodopsin, a naturally light-sensitive GPCR, while retaining the intracellular loops that dictate coupling specificity to Gs, Gi or Gq proteins [29, 101]. Upon light illumination, retinal photoisomerization within the rhodopsin core induces conformational changes that mimic native ligand-induced activation [102, 103].

Representative systems, including Opto-β_2_AR and Opto-α_1_AR, have been extensively used to dissect second messenger kinetics and signaling bias between different G protein branches [29, 68, 74]. The millisecond temporal precision of optical activation enables the detailed quantification of desensitization and feedback kinetics, which are often obscured in traditional pharmacological assays [104].

Functional validation of these tools confirms their low basal activity in the dark, reversible activation upon light cessation and preservation of the intended G protein coupling specificity [76]. Proper membrane localization and topology are typically verified by fluorescence tagging or immunostaining [76, 105, 106].

Recent efforts toward red-shifted or long-wavelength variants of opsin-based chimeric GPCRs (or their functional analogues), together with engineered retinal analogs, are motivated by the desire to improve tissue penetration and extend wavelength programmability [66, 107, 108]. These red-shifted and NIR-tuned variants conceptually align with broader efforts in the field to extend optogenetic receptor control toward deep-brain or systemic applications with reduced phototoxicity [109, 110]. Compared with chemogenetic GPCR actuators, Opto-XRs, thus, uniquely support high-temporal-resolution dissection of G-protein signaling kinetics and bias, while maintaining native downstream coupling architectures [32, 76, 111].

### Light-controlled RTKs (primarily strategy III: intracellular oligomerization)

Optogenetic receptor tyrosine kinases (Opto-RTKs) primarily employ light-induced dimerization (Strategy III) to reconstruct the activation logic of native growth factor receptors [112]. By fusing photodimerizing modules (e.g. CRY2/CIBN, iLID/SspB) to the cytoplasmic kinase domains, light illumination induces receptor clustering, autophosphorylation and subsequent activation of downstream pathways such as MAPK, PI3K/Akt and PLCγ [81].

Light-controlled Trk constructs (such as Opto-iTrkB or Dr-TrkB) and light-inducible epidermal growth factor receptor (EGFR) or EGFR-clustering systems have been used to dissect how the strength and duration of receptor activation determine distinct cellular outcomes, including proliferation, differentiation and survival [113–115]. By applying light pulses of varying lengths, it is possible to quantitatively modulate downstream signaling amplitude and kinetics, enabling the study of dynamic “frequency coding” within growth factor pathways [112].

Functional validation involves demonstrating canonical phosphorylation profiles, minimal dark-state activity and reversible activation cycles [116]. Correct membrane localization, achieved through N-terminal transmembrane domains or lipidation motifs (e.g. myristoylation), is crucial for functionality and is confirmed via live-cell imaging [88].

These tools have spectacularly empowered our current knowledge of temporal coding mechanisms in processes such as neuronal plasticity and tissue regeneration. In particular, Opto-RTKs exemplify how Strategy III (oligomerization-based control) can be directly mapped onto developmental and regenerative contexts where growth factor pulse shape and duration are key determinants of cell fate [113].

### Optogenetically engineered ligand-gated ion channels (primarily strategy IV: photoallosteric modulation)

Optogenetic principles have been applied to ligand-gated ion channels to create hybrids that preserve native ligand sensitivity while placing gating under optical control [117, 118]. A major route is photoallosteric regulation, most robustly implemented using covalently attached, photoswitchable azobenzene cross-linkers or tethered ligands to reshape transmembrane movements and pore opening [118, 119]. In P2X receptors, such “optopharmacology” approaches enable reversible optical control of ATP-evoked currents and have been validated by patch-clamp electrophysiology and imaging, typically yielding light-evoked responses comparable to native ATP responses and with low dark-state leak [120]. An indirect alternative is to regulate channels via upstream optogenetic GPCR signaling: activation of Gi/o-coupled Opto-GPCRs releases Gβγ to open G protein-gated K channels (GIRK) channels (a well-established mechanism), as demonstrated with optogenetic G-protein activators [121]. These channel-based systems thereby extend Strategy IV (photoallosteric gating) to excitable membranes, bridging the gap between biophysical ion-channel control and native synaptic or neuromuscular transmission [122, 123].

### Cytokine and enzyme-linked receptors (primarily strategy I: extracellular dimerization)

Optogenetic principles have also been extended to cytokine and enzyme-linked receptor signaling [124, 125]. Although direct optical engineering of full-length cytokine receptors such as erythropoietin receptor (EPOR) or tumor necrosis factor receptors (TNFR) has not yet been widely realized, the concept of extracellular dimerization has been effectively implemented using light-responsive modules to recapitulate ligand-induced receptor clustering [41].

In these designs, red/far-red-responsive photodimerizers such as PhyB/PIF or CBCR pairs are anchored to receptor or adaptor scaffolds at the plasma membrane [126]. Illumination triggers reversible dimerization and the subsequent recruitment of downstream effectors, thereby mimicking cytokine-induced receptor proximity [127]. This approach allows optical reconstruction of signaling axes such as Janus Kinase/Signal Transducer and Activator of Transcription (JAK/STAT) and NF-κB, even when applied to synthetic or chimeric receptor frameworks rather than to native ectodomains [128].

An alternative route employs photopharmacological control using azobenzene-based photoswitchable ligands, which reversibly toggle between active and inactive conformations upon light exposure [129]. In principle, such ligands can modulate cytokine or growth-factor pathways without genetic modification, offering complementary temporal precision and compatibility with native receptor complexes [130, 131].

Among such approaches, the illumination intensity, duration or pulsing frequency could be varied for programmable tuning of cytokine-related signaling strength and dynamics [132, 133]. The optogenetic and photochemical systems hereby represent potent tools for spatiotemporally resolved modulation of immune and regenerative signaling pathways while preserving structural integrity and physiological logics of native cytokine networks [128]. Cytokine and enzyme-linked receptor designs, thus, exemplify Strategy I (extracellular dimerization), where receptor clustering geometry and duration are directly translated into immune or regenerative outcomes [134].

### Synthetic immune receptors: optogenetically controlled CAR-T systems

Building upon the same optogenetic engineering logic, these principles have also been successfully translated into cellular immunotherapy platforms, demonstrating their scalability from molecular to cellular levels [135]. While the previous sections addressed the optogenetic engineering of natural receptor scaffolds, these design logics can be transposed to synthetic immune receptor architectures, thereby expanding the definition of “optogenetic engineering receptors” from molecular systems to programmable cellular therapeutics [34].

The optogenetically controlled CAR-T cells represent the translation of this concept into a paradigm-shifting implementation designed to mitigate the “on-target, off-tumor” toxicity and cytokine release syndrome (CRS) well-documented in conventional CAR-T therapy [34]. These systems primarily utilize two distinct optical gating strategies, corresponding to the design paradigms outlined in Design Strategies for the Optogenetic Engineering of Receptors Section:

- Light-Induced Assembly of Split CARs (Strategy III): Systems such as LiCAR-T and other blue-light-regulated split-CAR designs, often referred to as optogenetically controlled chimeric antigen receptor T cells (OptoCAR-T) and hereafter termed OptoCAR, split the CAR into membrane-proximal recognition and intracellular signaling fragments, each fused to a photodimerizer (e.g. LOV2-SsrA/SspB, CRY2/CIB). Blue light drives reversible assembly of a functional CAR at desired times/locations, with quiescence in the dark [34].
- Optical Control of CAR Gene Expression (Strategy IV): The LINTAD system uses a light-inducible transcriptional circuit (CRY2-CIB1 mediated nuclear translocation/dimerization) to define time-gated CAR expression, acting as a safety switch that turns off upon light withdrawal [18].
- Wireless Deep-Tissue Activation: Upconversion nanoparticles (UCNPs) convert NIR to blue light in vivo, enabling wireless activation of LiCAR-T in murine lymphoma and melanoma models [34, 136].

A significant advancement for *in vivo* translation is the integration of UCNPs. These nanoparticles absorb deeply penetrating near-infrared light and emit localized blue light, thereby functioning as wireless mediators that activate LiCAR-T cells situated deep within tissues, as demonstrated in murine lymphoma and melanoma models [34].

In sum, optogenetically controlled CAR-T systems epitomize how design principles at the level of receptors can be scaled hierarchically—from molecular switches to functional therapeutic circuits—embodying in full the spirit of optogenetic receptor engineering and pointing to a blueprint for customized cellular therapies that will be controllable in space and time [137]. They, therefore, provide a compelling example of how optogenetic design strategies can be translated from receptor-level control to clinically relevant, programmable cell therapies [34].

## Applications of optogenetically engineered receptors

**Figure 3:**
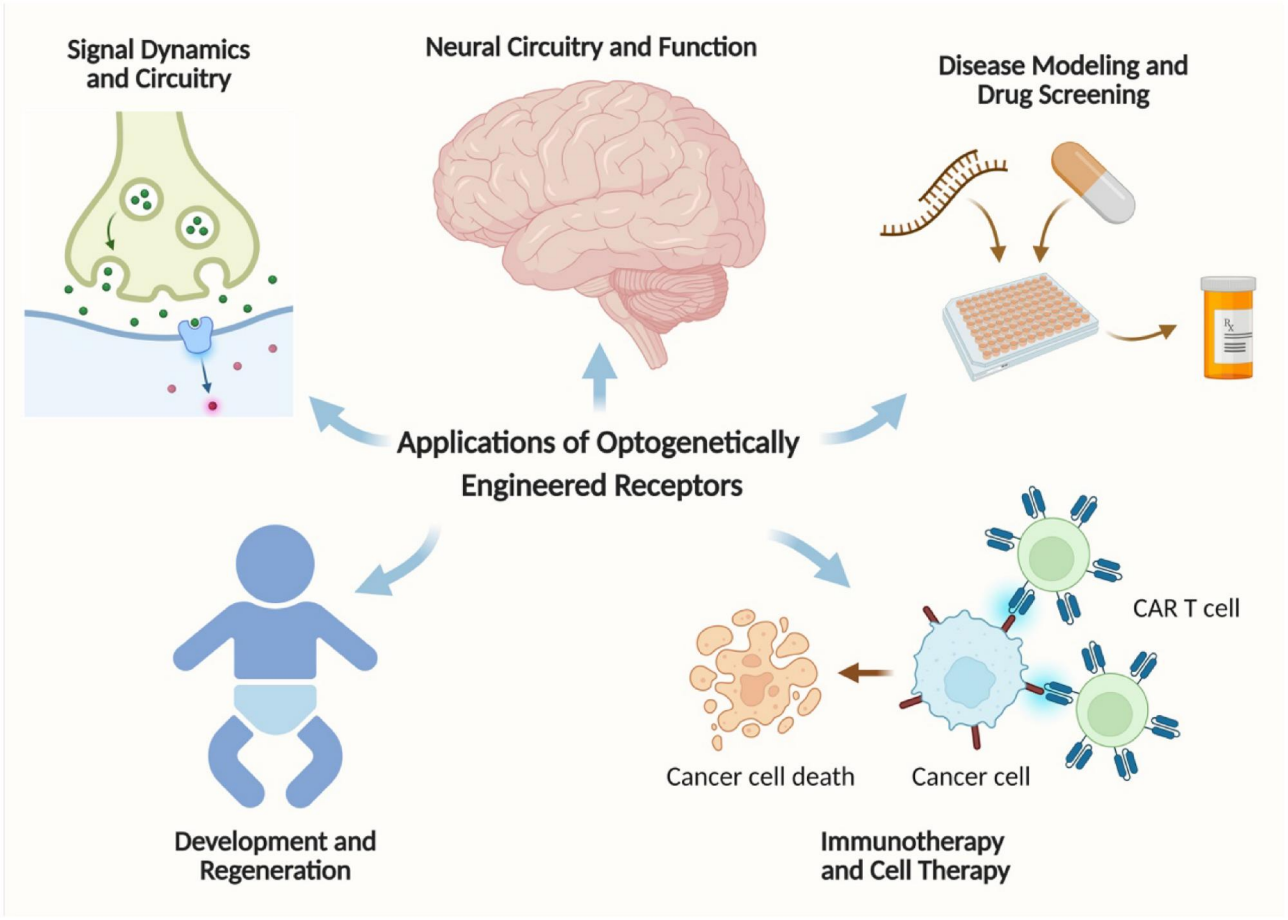
Translational landscape of optogenetic receptor engineering. Light-programmable receptors enable precise dissection of signal dynamics and circuitry, neural circuitry and function, disease modeling and development and regeneration, drug screening and reversible control of cellular immunotherapies such as optogenetic CAR-T cells.

Optogenetic engineering of receptors has transformed how receptor biology can be interrogated and manipulated across the scales of life [112]. These tools integrate precise optical control with endogenous receptor signaling, enabling investigation into the causal drivers of communicating dynamics, refined models of neural-immune regulation and introducing programmable paradigms for development and regeneration (Figure 3) [115, 133, 138–142]. These applications build directly upon the mechanistic design logic outlined in Design Strategies for the Optogenetic Engineering of Receptors Section, demonstrating how modular photosensory domains translate into function-specific receptor behaviors and ultimately tailored biological outputs [143]. A concise overview of how these design strategies map onto receptor classes and their corresponding biological outputs is summarized in Table 2.

**Table 2:** Overview of optogenetic receptor engineering strategies mapped to receptor classes and biological outputs.

Design strategy	Representative photosensory modules	Receptor classes targeted	Controlled outputs	Biological/Clinical applications
Strategy I: Extracelluar dimerization	PhyB/PIF, BphP1–QPAS1, CBCR pairs	RTKs, cytokine receptors	Receptor dimerization, clustering, JAK/STAT activation	Immune modulation, regeneration
Strategy II: Transmembrane/Rhodopsin-based	Rhodopsins, melanopsin	GPCRs	Gi/Gs/Gq signaling, biased signaling	Neuromodulation, behavior control
Strategy III: Light-induced oligomerization	CRY2, CRY2olig, iLID/SspB	RTKs, CARs	MAPK/ERK, PI3K/Akt, immune synapse formation	Cell fate control, CAR-T tuning
Strategy IV: Photoallosteric regulation	LOV2 insertion, azobenzene photoswitches	Ion channels, enzyme-linked receptors	Conformational gating, pore opening/closing	Optopharmacology, circuit dissection

### Decoding receptor signaling dynamics

In fact, optogenetically engineered receptors revolutionized the quantitative study of receptor signaling by offering, for the first time, complete control over timing, intensity and spatial locale of receptor activation. This enables moving beyond correlative observations toward causal dissection [53].

For instance, light-pulse protocols in studies of GPCR allow the exact measurement of the desensitization, endocytosis and resensitization kinetics with subsecond resolution, parameters convoluted in traditional pharmacology [29]. Further, the latter approach was instrumental to establish that encoding of signaling intensity and duration directly dictates downstream transcriptional and metabolic outcomes, formalizing a principle of “signal encoding” in cellular decision-making [60, 144, 145].

Similarly, pulsed illumination in optogenetic RTK systems, such as light-inducible FGFR1 (OptoFGFR1) and Trk constructs (e.g. Opto-iTrkB or Dr-TrkB), could generate controlled oscillations in MAPK and PI3K/Akt activity, which might reveal how specific temporal patterns of pathway activation drive distinct cellular outcomes, including proliferation, differentiation or apoptosis [146].

By decoupling receptor activation from native ligand binding, optogenetic control effectively converts signaling dynamics into a programmable input language, enabling systematic mapping of causal links between molecular-level timing and cell-fate decisions [60]. In contrast, chemogenetic systems—due to slow ligand diffusion, receptor occupancy kinetics and limited reversibility—cannot support oscillatory paradigms or fine-tuned temporal encoding, underscoring the unique role of optogenetic receptors in dissecting fast signaling dynamics [104, 147].

### Neuroscience: input–output decoupling and glia-neuron interplay

In neuroscience, genetically engineered optogenetic receptors go beyond the optical control of electrical firing provided by channelrhodopsins by enabling optical interrogation of biochemical signaling [32, 104, 145, 148].

Opto-β_2_AR and other light-controlled GPCRs may enable the specific neuromodulatory pathways, such as adrenergic or dopaminergic, to be turned on without direct changes in membrane potential, and thus, directly and selectively control intracellular cascades involved in synaptic strength and circuit plasticity [29, 149]. Such mechanistic precision is difficult to achieve with chemogenetic DREADDs, which exhibit slower onset, broad diffusion of ligands and poor spatial confinement, limiting their utility for dissecting rapid neuromodulatory events or glia–neuron dynamics [30].

This capability is crucial for distinguishing the role of metabotropic signaling from fast ionotropic transmission, functionally decoupling the biochemical “input” from the electrical “output” of a neuron [149, 150]. Moreover, these tools are equally powerful in glial cells. Consequently, through optogenetic control of purinergic or chemokine GPCRs, it is possible to adroitly manipulate gliotransmission and neuroinflammatory signaling *in vivo* [151]. This is a means for reversible, cell-type-specific interrogation of astrocyte-neuron and microglia-neuron communication of its functional basis in the coordination of these cell types in brain homeostasis, circuit refinement and disease states [152]. In combination with traditional excitatory optogenetic tools, these receptor-based systems enable comprehensive, bidirectional modulation of neural network function-delivering both direct stimulation and nuanced metabotropic modulation with light-defined precision [149].

### Disease modeling and drug discovery

Optogenetic receptor engineering provides a powerful strategy for modeling pathogenic receptor signaling states and for validating novel therapeutic targets with high causal inference [116, 153].

This enables the light-dependent, reversible activation of selected GPCR or RTK pathways and, consequently, the direct testing of how aberrant, sustained signaling contributes to pathogenesis in disorders such as Parkinson’s disease, depression and cancer [29]. For instance, precise activation of dopaminergic or serotonergic pathways through Opto-GPCRs allows mechanistic studies of reward processing, motivation and mood regulation in behaving animals [29].

In drug discovery, these engineered receptors serve as optically gated, high-fidelity screening platforms. Unlike static ligand-binding assays, they recapitulate the dynamic nature of physiological receptor activation [154], enabling the stimulation of signaling with precise timing and amplitude to discriminate candidate drugs based on their effects on key processes such as receptor desensitization, internalization and importantly, biased signaling, where a given drug preferentially activates one downstream pathway over another [112].

Thus, optogenetic receptor systems bridge the gap between high-throughput screening and physiological relevance of the cell, hence, very important in advancing toward predictive pharmacodynamics.

### Development and regeneration

**Figure 4:**
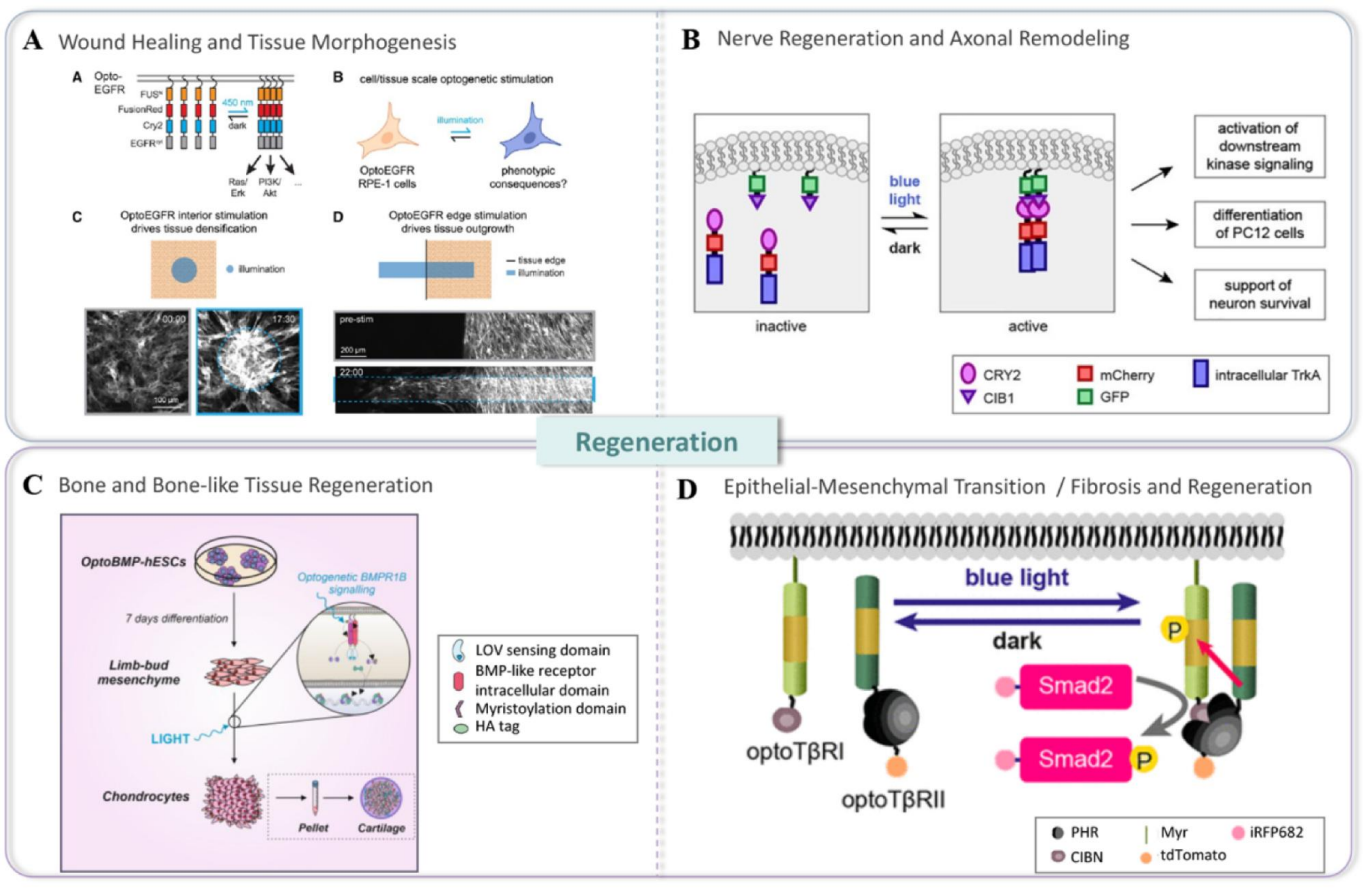
Applications of optogenetically engineered receptors in development and regeneration. Applications of optogenetically engineered receptors in (**A**) wound healing and tissue morphogenesis (reprinted from Suh *et al*. with permission from Elsevier) [155], (**B**) nerve regeneration and remodeling [81], (**C**) bone and bone-like tissue regeneration (reprinted from Humphreys *et al*. with permission from American Chemical Society) [156] and (**D**) epithelial–mesenchymal transition (EMT)/fibrosis and regeneration (reprinted from, Li *et al*. with permission from American Chemical Society) [157].

As such, optogenetically controlled growth factor and cytokine receptors are opening completely new frontiers in spatially and temporally precise tissue engineering and regenerative medicine (Figure 4) [155]. These examples demonstrate how modular design features—such as light-induced dimerization or LOV2 allosteric gating—directly map onto tissue-level patterning behaviors. In epithelial tissues, the optogenetic epidermal growth factor receptor (OptoEGFR) enables millimetre-scale control of collective cell rearrangements and edge outgrowth, driven predominantly by PI3K rather than ERK signaling-a powerful route to program wound closure and morphogenesis [155].

The techniques of optogenetic tropomyosin receptor kinase B (Opto-TrkB) and optogenetic fibroblast growth factor receptor (Opto-FGFR) allow the differential direction of *in vitro* stem-cell differentiation and morphogenetic organization with unprecedented precision by inducing signaling pathways via patterned illumination [81, 146]. And OptoEGFR extends that precision to tissue-level collective dynamics. This opens up perspectives for realizing synthetic, light-defined morphogen fields that mimic developmental cues with the unprecedented option for real-time, feedback-controlled perturbation.

In regenerative biology, optical activation of RTKs or cytokine receptors offers the potential to synchronize pro-repair signaling with specific stages of the healing process or local microenvironmental states, thereby promoting controlled angiogenesis, axonal regrowth or tissue remodeling [31, 133, 141, 158, 159]. Consistent with this, OptoTrkB drives PI3K/Akt and Raf/ERK activation to enhance neurite outgrowth in PC12 cells and support DRG neuron survival even in the absence of NGF [81, 160]. For skeletal and cartilage contexts, light-responsive BMP receptor systems enable rapid and reversible activation of SMAD1/5/8, providing a dose-by-time programmable handle for osteo-/chondrogenic regeneration [159]. Moreover, optogenetic TGF-β receptor pairs (OptoTGFBRs) allow single-cell-resolution pulsing or sequential activation to interrogate and steer EMT, fibrosis and lineage decisions during repair [157, 161].

The integration of optogenetically engineered cells with programmable, photoresponsive biomaterials (e.g. hydrogels that release factors upon light exposure) further enhances our ability to construct artificial signaling niches that can dynamically support and guide long-term tissue regeneration and repair [162]. Together, these approaches illustrate how optogenetic receptor engineering can emulate natural morphogen gradients and coordinate multicellular patterning with unprecedented precision.

### Precision immunotherapy and cellular therapies

The concept of optogenetic receptor engineering has recently been extended to adoptive cell therapy, particularly CAR-T cell therapy, to address its major safety limitations such as on-target/off-tumor toxicity and CRS [163]. By integrating optical control modules into CAR-T constructs, researchers have developed systems like LiCAR-T, OptoCAR and LINTAD-CAR, which enable the activity of therapeutic cells to be precisely regulated by light [18, 34, 89]. In these designs, light-induced dimerization or transcriptional control circuits are used to restrict CAR activation or expression to defined spatiotemporal windows, ensuring that T-cell cytotoxicity occurs only within illuminated tumor regions [18, 164]. This optical gating conceptually confers several advantages: it spatially restricts immune activation to specific anatomic sites, permits stratification of therapeutic intensity via light dose or duration modulation, and is theoretically reversible by simple cessation of illumination [34, 165]. Recent combinations with UCNPs have achieved true remote and nearinfrared light-driven control over the activation of CAR-T cells deep within target tissues, effecting tumor eradication with substantially reduced systemic toxicity in preclinical models [18]. Collectively, these optogenetically engineered CAR-T approaches illustrate how receptor level design principles can be hierarchically scaled to confer safety, programmability and reversibility on cellular immunotherapies and have formed a key translational milestone for the field of optogenetic receptor engineering in precision medicine.

## Advantages, challenges and optimization strategies

### Advantages

Optogenetically engineered receptors combine the physiological fidelity of native signaling with the temporal precision and reversibility of optical control [68]. Unlike fully synthetic receptors, they retain endogenous coupling to second-messenger pathways and regulatory feedback, preserving biological realism [113]. Their reversible activation-deactivation cycles enable real-time modulation at subcellular and millisecond scales, surpassing chemical or genetic perturbations in both precision and reproducibility [166]. Moreover, the optical input is noninvasive, multiplexable and orthogonal to biochemical noise, allowing simultaneous interrogation of multiple receptor pathways within a single cell or tissue context [82, 150].

### Challenges

With such versatility, however, it turns out that several challenges remain before the performance of optogenetic receptor systems can be robust and translational.

- Dark-state leakage and basal activity. This arises because of the imperfect folding or incomplete shielding of the chromophore; many engineered receptors exhibit low-level spontaneous signaling [32, 167].
- Sensing bias and distortion: The fusion of exogenous photodomains could affect G-protein or kinase coupling, thereby making it differ from the receptor’s natural response spectrum [32].
- Intended folding and localization: Large domains of photoreceptors might interfere with membrane trafficking or topology; special optimization of the linkers is necessary [21, 70].
- Cofactor and lighting restrictions: Many photoreceptors, such as phytochromes and CBCRs require exogenous chromophores like PCB, which limit *in vivo* applicability [21, 64].
- Delivery and immunogenicity—efficient gene delivery and immune compatibility remain major bottlenecks to translational use in mammals [38, 168].

Additionally, long-term biosafety considerations—including chromophore stability, dark-toxicity, immune memory and the immunogenicity of non-human photoreceptors—pose significant hurdles for clinical translation [169, 170]. Efficient and safe delivery, especially through nonviral carriers (e.g. lipid nanoparticles, mRNA delivery or polymeric vectors), as well as autologous cell engineering, may mitigate these concerns and improve translational feasibility [171, 172]. Collectively, these challenges define the performance ceiling for optogenetic receptor systems and point toward successive engineering refinements.

### Optimization strategies

To overcome these limitations, the focus of current efforts is on rational structural design, directed evolution and orthogonal optical control [21, 82]. Careful optimization of linker length, fusion topology and insertion sites is required [53]. This is approached by structure-guided modeling with the aim of maintaining proper protein folding and preserving coupling integrity. In addition, strategies of directed evolution are pursued [173]. Strategies are combined with high-throughput optical screening to select variants with improved stability, reduced leakiness and enhanced dynamic range [174]. Orthogonal and multicolor systems, including those based on CRY2/CIB, CBCR and redshifted opsins, enable parallel modulation of distinct receptor pathways free of spectral interference [175, 176]. Emerging approaches such as machine-learning-guided protein engineering and AI-driven structural prediction may accelerate the identification of optimal fusion topologies, LOV insertion sites and linker configurations that minimize leakiness while maximizing photoswitching efficiency [177, 178].

Looking forward, the next frontier in optimizing optogenetic control may lie in cross-disciplinary integration with bioengineering concepts. For instance, the principle of signal amplification embodied in nanostructure-gated organic electrochemical transistors (OECTs), which enhance weak bioelectrical signals through transconductance, could inspire the design of future optogenetic systems with built-in signal gain modules [179]. Similarly, strategies to improve signal-to-noise ratio, such as the interfacial coating approaches used in magneto-fluorescent probes to boost optical stability and sensitivity [180], offer valuable paradigms for engineering optogenetic tools with lower background activity and higher fidelity in complex tissue environments. While these are currently device-level concepts, they highlight a promising direction: translating engineering principles from bioelectronics into molecular design to create a new generation of high-performance optogenetic receptors.

Ultimately, establishing standardized validation frameworks, scalable manufacturing workflows and clinically compliant illumination/delivery systems will be key to transitioning optogenetic receptors from experimental tools toward therapeutic-grade technologies.

## Conclusion and perspectives

By combining precise optical control with the inherent complexity of native biological signaling, design of optogenetic receptors sets a paradigm for interrogating and effectively controlling cellular communications. Thus, with their approach, by installing genetically encoded optical switches onto natural receptor frameworks, this really transformed the dissection of signaling dynamics with spatiotemporal resolution that was previously unattainable, bridging the critical gap between observational biology and causal intervention. These advances illustrate how optogenetic receptor systems uniquely preserve endogenous signaling logic while introducing programmable, reversible and quantitative external control—an attribute rarely achieved with chemical, genetic or mechanical modalities.

It will now forge ahead—from the development of basic tools to integrated physiological and therapeutic applications. Essential frontiers include the translation to deep-tissue contexts through novel approaches in near-infrared photoreceptors, implantable micro-optoelectronics and bioluminescent resonance; the development of spectrally orthogonal multiplexed systems for the parallel independent control of many pathways to achieve the systematic deconstruction of complex cellular networks; and standardized design rules and rigorous preclinical validation pipelines are essential to ensure the safety, reliability and eventual clinical translation of such technologies. Clinical translation will further depend on overcoming immunogenicity of non-human photodomains, improving gene-delivery efficiency and ensuring long-term biosafety. Nonviral delivery (e.g. mRNA or nanoparticle vectors) and autologous cell engineering represent promising solutions for clinical-grade deployment. In parallel, AI-guided protein engineering, machine-learning-based linker optimization and soft photonic biomaterials may accelerate the creation of next-generation optogenetic receptors with improved stability, spectral tunability and deep-tissue compatibility.

In all, the optogenetic receptor engineering imposes a common blueprint for the next generation of precision biomedicine. Such work takes the center stage on how synthetic biology can master and execute, rather than rewrite, nature’s logic. This approach opens up possibilities for a new generation of receptor-based therapies that are reversible, programmable and highly specific.

Altogether, these developments underscore a realistic pathway for transitioning optogenetic receptors from foundational tools to therapeutic-grade systems capable of achieving spatiotemporally resolved interventions in regenerative medicine, immunotherapy and neuromodulation.

## Fundings

This work was supported by the Science and Technology Program of Zhejiang Province (grant number: 2024C03194) (to G.Y.), the National Natural Science Foundation of China (grant number: 82301018) (to W.C.), the Foundation of Muyuan Laboratory (grant number: 13166022401) (to J.Y.), the National Key Research, Development Program of China (grant number: 2024YFA1107800) (to J.Y.), the National Natural Science Foundation of China (grant number: 32501207) (to Z.G.), the National Natural Science Foundation of China (grant number: 52403205) (to L.Z.), China Postdoctoral Science Foundation funded project (grant number: 2025M772889) (to Z.G.).
